# True Prevalence and Seroprevalence of Piroplasmosis in Horses in Southwestern Europe

**DOI:** 10.3390/ani15142047

**Published:** 2025-07-11

**Authors:** Juan Duaso, Alejandro Perez-Ecija, Ana Navarro, Esther Martínez, Adelaida De Las Heras, Francisco J. Mendoza

**Affiliations:** 1 Department of Animal Medicine and Surgery, University of Cordoba, 14014 Cordoba, Spain; juanduaso@gmail.com (J.D.); v02hesaa@uco.es (A.D.L.H.); fjmendoza@uco.es (F.J.M.); 2 Gasset Laboratory, DAV Salud Group SL, 18200 Granada, Spain; ana@grupodavsalud.es (A.N.); esther@grupodavsalud.es (E.M.)

**Keywords:** Andalusia, *Babesia caballi*, PCR, regionality, seasonality, serology, *Theileria equi*

## Abstract

Equine piroplasmosis (EP) is an endemic parasitic disease in southern European countries, such as Spain. Andalusia, the most southwestern region of Europe, is the community with the highest number of registered horses and farms in Spain and one of the main international exporters of Andalusian (Spanish Purebred) horses worldwide. Considering the current expansion of this disease and the possible effect of climate change on its prevalence, studying the EP prevalence in this region is compelling. Molecular (PCR) and serological methods (cELISA and IFAT) were used to study the true and apparent prevalences during a period of three consecutive years, evaluating the effects of age, sex, season, year of testing, and province. Results showed different EP prevalences between western and eastern provinces, as well as among seasons. Moreover, a positive association was observed between age and *T. equi* seropositivity, without any effect of sex. Our findings demonstrate that Andalusia is an EP endemic region, but prevalences were lower compared to central and northern Spanish regions. Moreover, EP prevalence has not increased in Andalusia in recent years despite climate changes.

## Simple Summary

Equine piroplasmosis (EP) is a parasitic tick-borne disease caused by *Apicomplexa hemoprotozoans* with a worldwide distribution. Due to global warming, tick expansion to previously EP-free countries, and higher international horse movements, the prevalence of this disease is increasing worldwide. This study evaluates EP prevalences by PCR and serology in Andalusia, the most southwestern European region and one of the main international horse exporters. Results showed regionality (with different EP prevalences between western and eastern provinces), seasonality, and age effects (old horses more likely to be seropositive), respectively. Although Andalusia is an EP endemic region, an increase in EP prevalences was not observed during the studied period.

## 1. Introduction

Piroplasmosis is a tick-borne disease that can affect any equid (horses, donkeys, mules, and zebras) caused by protozoans from the genus Apicomplexan (*Babesia caballi* and *Theileria equi*) and transmitted by hard ticks from the genus Ixodes [1]. Recently, a third emerging causative agent has been described (*Theileria haneyi*) with limited presence in some countries, such as the USA, Italy, China, and South Africa, among others [2,3,4,5].

Equine piroplasmosis (EP) is linked to serious commercial, economical, and sanitary implications for equids worldwide. This disease limits equid international sales and movement to events such as sport competitions, auctions, or shows, also causing high economic losses due to veterinary costs, abortions, deaths, or poor performance [6,7].

Historically, EP has been more common in warm locations, such as Africa, South and Central America, and the Mediterranean basin [1]. However, due to global warming and the subsequent expansion of vector ticks to colder regions, EP has spread towards previously free regions in north and eastern Europe [8], now even considered endemic in some of these countries (e.g., Germany) [9]. Because of the emergence of EP in these previously free regions, this disease is now considered in sick horses with unspecific signs such as fever, icterus, malaise, and poor performance in these countries [10].

EP is an endemic disease in Spain due to its warm climate [11]. Spain, sited in the south of Europe, is one of most important international exporters of horses (especially the Spanish Purebred, also named the Andalusian breed), mainly to the USA, Mexico, and South America (e.g., Colombia, Ecuador), as well as to the rest of Europe. Andalusian horses can be transported freely within the Schengen area, which could lead to the movement of EP infected horses without any restriction throughout Europe. *T. haneyi* has not been reported in Spain at this moment.

Andalusia (approx. 87,000 km^2^) is the southernmost region of mainland Europe and Spain (37°24′18″ N 5°59′15″ O/37.405, −5.9875) and is, by far, the region with more registered horses (32.3%) and equine farms (39.0%) than others in Spain [12]. The most common breed (70%) in Spain is the Andalusian breed, and most Andalusian horses and farms are based in Andalusia [12]. More than ten years ago, two studies evaluated the seroprevalence of EP in Andalusia [13,14], showing remarkably high seroprevalences for *T. equi* (43–50%), *B. caballi* (11–22%) and co-infection (8–15%) in horses [13,14]. Another more recent study, compiling data obtained both by PCR and serology from all over Spain, reported different prevalences [15]. Despite these previous reports, and in the face of the changing epidemiology of EP due to global warming, as well as considering the importance of Andalusia for the equid industry and from a geographical point of view, it is compelling to update the prevalence data (based both in PCR and serology) of EP in this region.

Therefore, the aims of our study were to update the true prevalence and seroprevalence of EP in horses residing in Andalusia and to evaluate the effect of several contributors, such as year, season, age, and gender, on these prevalences.

## 2. Materials and Methods

### 2.1. Study Design

All blood samples submitted for EP analysis, either by PCR or by serology, to a national private veterinary reference laboratory (Gasset Laboratory, DAV Salud Group SL, Granada, Spain) from horses residing in Andalusia during a three-year period (from January 2022 to December 2024) were retrospectively selected from a database.

Samples with lipemia, hemolysis, or any abnormal sample processing (e.g., long storage, shipping delay longer than 24 h, clotted sample, etc.) were discarded. No repeated analyses from the same horse were included. Blood samples from donkeys and mules were excluded from the study.

The total number of blood samples (897) was calculated using the formula for a finite population, taking into consideration the number of horses registered in Andalusia during the previous year 2024 [16]. The number of horses to be sampled from each district (province) within Andalusia (Table 1) was also determined according to the livestock census provided by the Andalusian government in 2024 [16]. The conditions for the sample calculation were fitted to ±3% precision, a 95% confidence interval (CI), and an expected prevalence of 30% [15] for *B. caballi* and *T. equi*.

The minimum number of blood samples (897) was calculated using the formula for a finite population, taking into consideration the number of horses registered in Andalusia in 2024 (159,038), a 95% confidence interval, a 3% of maximum error, and an expected prevalence of 30% for both parasites.

Information on breed, sex, age, and date of submission was retrieved from the database to evaluate the effects of these variables on EP results. No information on clinical signs was included in the statistical analysis in order to avoid any bias. Seasonality was defined as spring (March–May), summer (June–August), autumn (September–November), and winter (December–February).

### 2.2. Seroprevalence Study

#### 2.2.1. Competitive Enzyme-Linked Immunosorbent Assay (cELISA)

Two WHOA-approved equine commercial cELISA kits (*T. equi* or *B. caballi*, VMRD Inc., Pullman, WA, USA) [17] were used to analyze all of the serum samples within 48 h of submission. Sensitivity and specificity, according to the manufacturer, are 95% and 99.5% for *T. equi* and 100% and 100% for *B. caballi*. The same operator performed every assay following the manufacturer’s protocols. A microplate reader (ChroMate 4300, Awareness Technology Inc, Palm City, FL, USA) was used to read optical densities at 630 nm. Horses were considered positive, both for *T. equi* and *B. caballi*, when the inhibition percentage was higher than 40%.

#### 2.2.2. Indirect Fluorescent Antibody Test (IFAT)

Two validated commercially available equine kits (MegaFLUO^®^ *T. equi* or *B. caballi*, Megacor Diagnostik, Hörbranz, Austria) [18] were used to analyze the presence of IgG against *T. equi* or *B. caballi* in serum samples within 48 h of submission. Briefly, serum samples were diluted from 1:80 to 1:280 in phosphate-buffered saline (PBS). Pre-fixed slides were first incubated with 20 µL of serum at 37 °C for 30 min, followed by three washes with PBS, and a second 30 min incubation with 20 µL of FITC anti-horse IgG conjugate. After washing with PBS, fluorescence was evaluated using a trinocular fluorescence microscope (Balea 8100, Beortek SA, Vizcaya, Spain). Positive and negative controls were included in each assay. The cut-off for seropositivity, for both parasites, was a fluorescent signal greater than 1:80. The exact sensitivity and specificity of this test are unknown, but this technique is considered a highly specific confirmatory test [19].

### 2.3. True Prevalence Study (PCR)

DNA was extracted from 200 µL EDTA blood using an automatized nucleic acid extraction system (MagNA Pure 24 Instruments, Roche Diagnostics, Barcelona, Spain) following the manufacturer’s instructions [20]. Extracted DNA was frozen at −20 °C until real-time qPCR was performed.

A singleplex real-time PCR was performed using commercial DNA *T. equi* or *B. caballi* detection kits (genetic PCR solutions, Alicante, Spain) in an automatized thermocycler (LightCycler 480, Roche Diagnostics, Barcelona, Spain). These kits contain individual ready-to-use tubes containing all the components needed to perform the PCR (mastermix, primers, DNase/RNase-free water, etc.). Internal, negative, and positive controls were also provided by the manufacturer in the same kits. The PCR conditions, in a final reaction volume of 20 µL (5 µL of DNA) for both parasites, were an initial activation at 95 °C for 2 min, followed by 40 cycles of 5 s at 95 °C (denaturation), and a final step at 60 °C for 20 s (hybridization/extension).

### 2.4. Statistical Analysis

The true prevalence and seroprevalence of *B. caballi* and *T. equi* in Andalusia and in each province within Andalusia were calculated using WinEpi 2.0 software (Working in Epidemiology, Zaragoza, Spain). The rest of the analyses were performed using a specific statistical package (IBM SPSS Statistics 27, IBM Corporation, Armonk, NY, USA).

When applicable, results are expressed as means ± standard deviations (SDs) or medians and interquartile ranges (IQRs, 25th–75th percentiles), according to the distribution. The Kolmogorov–Smirnov test was used to assess normality. Differences between groups were calculated using a Chi-square test. In addition, Spearman’s or Pearson’s coefficients were used to determine correlations between parameters as appropriate. Values with *p* < 0.05 were considered significant.

## 3. Results

The recommended size of horse samples was 897 from a total of 159,038 horses registered in Andalusia in 2024 (Table 1). This number corresponds to 36% of the total registered horses in Spain [12]. A total of 1182 horses were initially evaluated, with 1002 samples fitting the inclusion criteria (105 samples more than the recommended number). The number of horses sampled from each province (eight districts) within Andalusia is compiled in Table 1. Four provinces (4/8, 50%) exceeded the recommended percentage of sampling: Cadiz, 187 horses (18.7%); Granada, 85 (8.5%); Malaga, 203 (20.3%); and Sevilla, 335 (33.4%). In contrast, we received a lower number of samples from the rest of the provinces (Table 1).

Regarding the studied population, the median age was 5.0 (4.0) years old, with 25.1% mares and 74.9% males, and 66.3% of the values missing. The most represented breed was the Andalusian (75.7%), followed by crossbred horses (14.5%), the Lusitano breed (3.8%) and the Spanish Sport Horse breed (CDE, 1.5%). Other breeds with less than 1% were KWPN, Arabian, Anglo-Arabian, Hispano-Arabian, Argentine Polo, Frisian, Westphalian, French Trotter, Zangersheide, Belgian Warmblood, etc.

### 3.1. True Prevalence of EP, B. caballi, and T. equi in Andalusia

Considering both PCR and serology results, 23.7% (95% CI: 21.0–26.3%) of horses were EP = positive in Andalusia. The true prevalence of *T. equi* (27.0%, 95% CI: 22.0–32.0%) in Andalusia was higher than that of *B. caballi* (4.7%, 95% CI: 2.3–7.1%), whereas 3.3% (95% CI: 1.3–5.4%) were PCR-positive for both parasites (co-infection) (Table 2).

**Figure 1:**
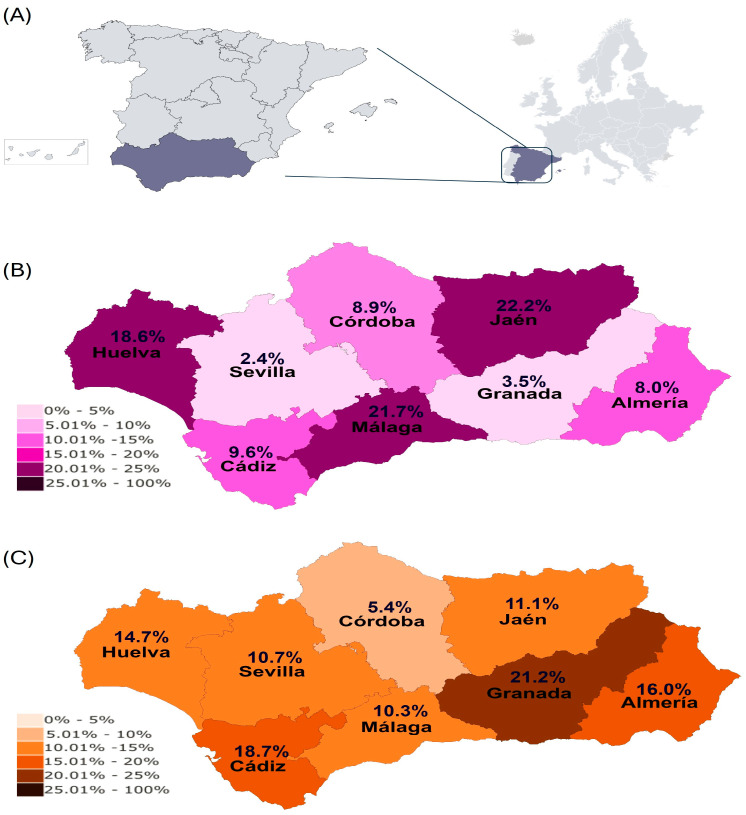
(**A**) Geographical location of Andalusia within mainland Europe. (**B**) True prevalence of EP in Andalusia. (**C**) Seroprevalence of EP in Andalusia.

Regarding the results based on the method for EP diagnosis, 101 horses were EP PCR-positive (10.1%, 95% CI: 8.2–11.9%), with Jaen (22.2%), Malaga (21.7%), with Huelva (18.6%) showing a higher true prevalence of EP than the rest of the provinces (Figure 1A).

**Figure 2:**
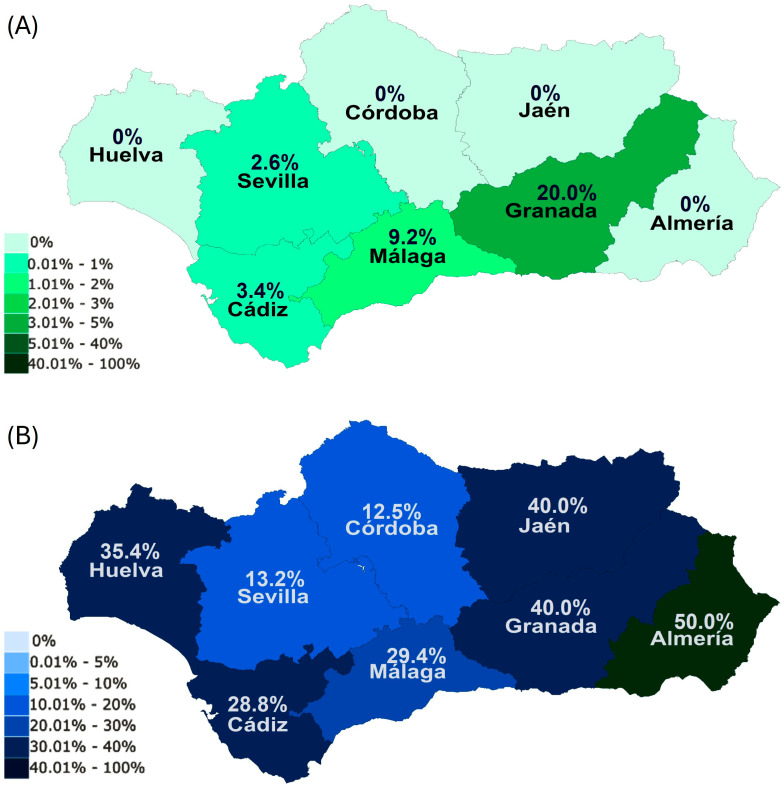
True prevalence levels of *B. caballi* (**A**) and *T. equi* (**B**) in each province within Andalusia.

Concerning the true prevalence within provinces for each parasite, Granada (20.0%), followed by Malaga (9.2%) were the provinces with the highest percentages of *B. caballi* PCR-positive horses, with the rest showing lower prevalences (Figure 2A). In contrast, Almeria (50.0%), Jaen (40.0%), and Granada (40.0%) were the areas with the highest true prevalences for *T. equi* (Figure 2B), with Sevilla (13.2%) and Cordoba (12.5%) having lower true prevalences than the rest of provinces for this parasite (Figure 2B).

### 3.2. Seroprevalence of EP, B. caballi, and T. equi in Andalusia

The seroprevalence of *T. equi* (14.3%, 95% CI: 11.8–16.9%) in Andalusia was higher than that of *B. caballi* (2.5%, 95% CI: 1.3–3.6), whereas 1.9% (95% CI: 0.9–2.9%) of horses were seropositive for both parasites (co-infection) (Table 2).

Regarding the results based on the method of EP diagnosis, 136 horses (13.6%, 95 CI: 11.5–15.7%) were EP-seropositive, with Granada (16.0%) and Cadiz (18.7%) being the provinces with higher EP seroprevalence (Figure 1B).

**Figure 3:**
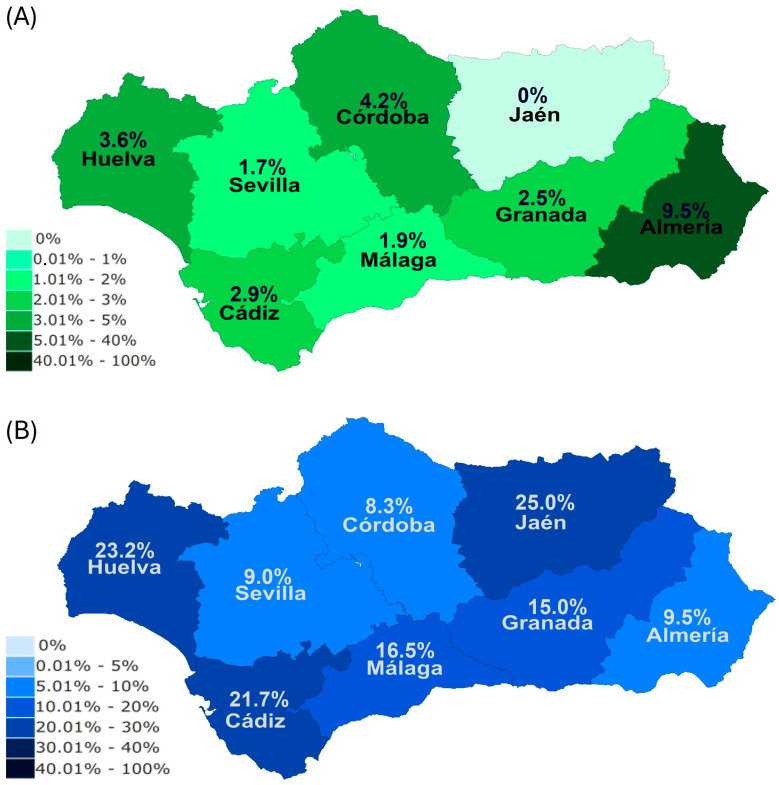
Seroprevalence levels of *B. caballi* (**A**) and *T. equi* (**B**) in each province within Andalusia.

Regarding the seroprevalence within provinces for each parasite, Almeria (9.5%) was the province with the highest seroprevalence for *B. caballi* (Figure 3A). In relation to *T. equi*, Jaen (25.0%), Huelva (23.2%), and Cadiz (21.7%) showed the highest seroprevalences (Figure 3B).

### 3.3. Effect of Contributors on True Prevalence and Seroprevalence of B. caballi and T. equi in Andalusia

#### 3.3.1. Season Effect

**Figure 4:**
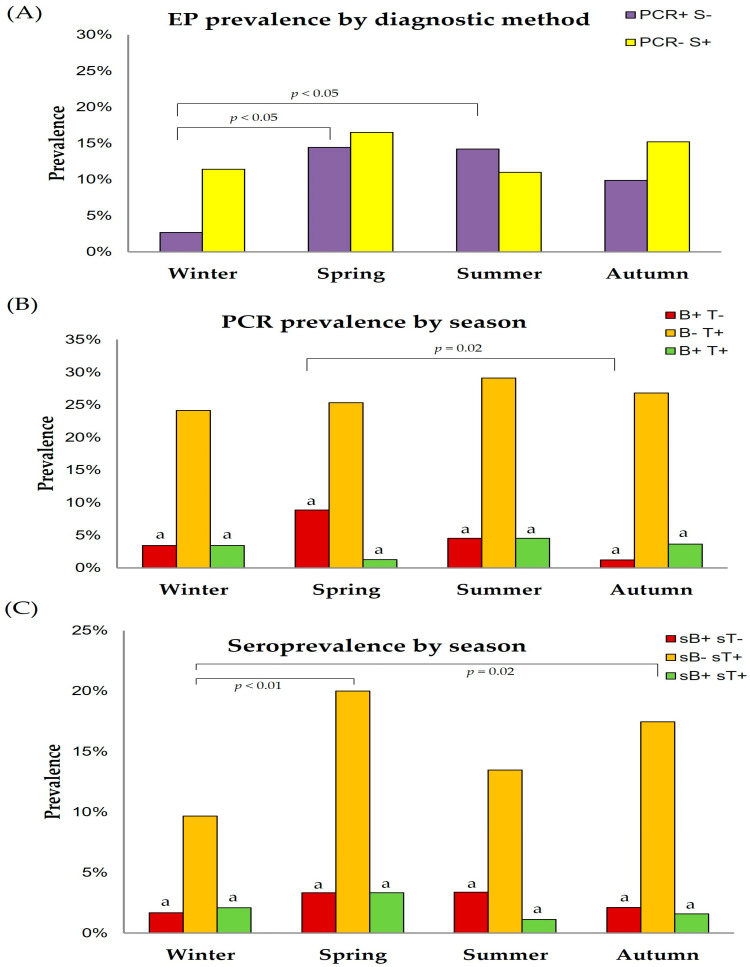
Effect of season on EP prevalence according to the used diagnostic method (**A**), on true prevalence levels (**B**) and seroprevalence levels (**C**). Brackets represent statistical differences between marked columns. B, *Babesia caballi*; PCR, polymerase chain reaction; S, serology; sB, *B. caballi* serology; sT, *T. equi* serology; T, *Theileria equi*. ^a^ *p* < 0.05 vs. *T. equi*.

The distribution of samples in each season was the following: 26.2% of samples submitted in winter, 19.4% in spring, 28.1% in summer, and 26.2% in autumn. Spring (14.4%, 95% CI: 9.5–19.4%) and summer (14.2%, 95% CI: 10.1–18.2%) were the seasons with the highest number of EP PCR-positive horses (PCR+ S−) in Andalusia, whereas EP PCR-positive results were significantly (*p* < 0.01) lower during winter (2.7%, 95% CI: 0.7–4.6%) (Figure 4A). No statistical differences were observed for EP-seropositive horses (PCR− S+) among seasons (Figure 4A).

Spring (8.9%, 95% CI: 2.6–15.1%) was the season with the highest *B. caballi* PCR-positive results, whereas autumn showed the lowest (1.2%, 95% CI: 0–3.6%) true prevalence for *B. caballi* (Figure 4B). In contrast, no differences were observed in the true prevalence of *T. equi* (B− T+) among seasons. Spring was the season with the lowest (1.3%, 95% CI: 0–3.7%) true prevalence of co-infection (B+ T+, Figure 4B), although statistical differences were not observed. In all seasons, the true prevalence of *T. equi* (24–29%) was statistically higher than that of *B. caballi* (<10%).

No significant differences were observed in the seroprevalence of *B. caballi* (sB+ sT−) and co-infection (sB+ sT+) among seasons (Figure 4C). In contrast, the seroprevalence of *T. equi* was higher in spring (20.0%, 95% CI: 12.8–27.1%) and autumn (17.5%, 95% CI: 12.1–22.9%) than in the rest of the seasons (Figure 4C). In all seasons, the seroprevalence of *T. equi* (10–20%) was significantly (*p* < 0.01) higher than that of *B. caballi* (<5%).

#### 3.3.2. Intra- and Interannual Effect of Testing

**Figure 5:**
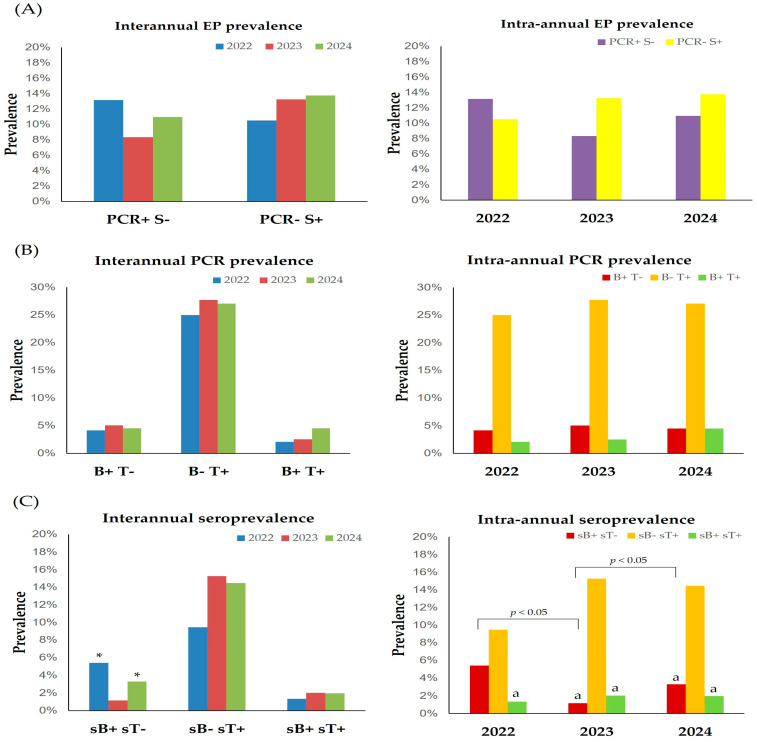
Effect of year of testing on EP prevalence according to the used diagnostic method (**A**), on true prevalence levels (**B**) and seroprevalence levels (**C**). Brackets represent statistical differences between marked columns. B, *Babesia caballi*; PCR, polymerase chain reaction; S, serology; sB, *B. caballi* serology; sT, *T. equi* serology; T, *Theileria equi*. ^a^ *p* < 0.05 vs. *T. equi*; * *p* < 0.05 vs. 2023.

Although a lower number of samples for EP diagnosis was submitted in 2022 (114) compared to 2023 (459) and 2024 (429), no differences were observed in the true prevalence or seroprevalence among years (interannual effect, Figure 5A). Regarding the intra-annual variability in Andalusia, the number of EP-seropositive (PCR− S+) horses was significantly higher than that of PCR-positive ones (PCR+ S−) in 2023 (13.2% versus 8.3%, respectively), but no differences were observed in 2022 (10.5% versus 13.2%, respectively) or 2024 (13.8% versus 11.0%, respectively) (Figure 5A).

Concerning PCR results, no significant interannual differences were observed for any of the groups (Figure 5B). PCR prevalence of *T. equi* was significantly (*p* < 0.05) higher than for *B. caballi* and co-infection in all the years studied (Figure 5B). Although the true prevalence of co-infection increased in 2024 (4.5%, 95% CI: 1.0–8.0%) compared to 2022 (2.1%, 95% CI: 0–6.1%) and 2023 (2.5%, 95% CI: 0–5.3%), no statistical differences were noted.

The seroprevalence of *T. equi* was higher in 2023 (15.3%, 95% CI: 11.5–19.0%) and 2024 (14.5%, 95% CI: 10.5–18.4%) compared to 2022 (9.5%, 95% CI: 2.8–16.1%) (Figure 5C). *B. caballi* seroprevalence was significantly (*p* < 0.05) lower in 2023 compared to 2022 and 2024 (Figure 5C). *T. equi* seroprevalence was significantly higher (*p* < 0.05) than that of *B. caballi* in 2023 and 2024 and than co-infection every year (Figure 5C).

#### 3.3.3. Gender and Age Effects

No differences between mares and males were observed in either the PCR prevalence or seroprevalence of any of the EP-causative agents, including co-infection. A higher (*p* < 0.05) number of horses were EP-seropositive compared to EP PCR-positive, both in mares and males.

Gender was not correlated with a higher likelihood of being PCR-positive or seropositive for *B. caballi* or for *T. equi* (Table S1). In contrast, age had a significant positive correlation with an EP-seropositive diagnosis (*p* = 0.025, ρ = 0.29) and with *T. equi* seropositivity (*p* = 0.015, ρ = 0.14) (Table S1).

## 4. Discussion

EP is endemic to Spain due to the warm climate, mainly in the south of the country, where wet and warm weather allows vectors to be present along the entire year. Andalusia is the most important region for the Spanish equid industry [12], with the highest number of registered horses and the highest number of farms of Andalusian horses, the main breed in Spain and its most exported breed worldwide. This reason, along with the geographical location of Andalusia at a continental crossroads and the impact of climate change in EP, calls for an updated EP prevalence study in this region.

EP has been previously studied in Andalusia in two reports, but blood samples were collected before 2014 [13,14]. Taking into consideration the dynamic and changing behavior of this disease due to global warming, an updated study of the disease in this region is compelling. In addition, only seroprevalence was studied in these previous reports, using either a cELISA or IFAT. Thus, this is the first study evaluating the true prevalence (molecular detection by PCR) of EP in Andalusia and comparing the results with seroprevalence by combining both cELISA and IFAT techniques.

Previous studies showed a total EP seroprevalence of 50.9 and 53.3% [13,14], both results being higher than in our study (13.6%), even considering the total EP prevalence combining both diagnostic methods (23.7%). Similarly to these studies, our results also showed higher seroprevalence of *T. equi* compared to *B. caballi*. However, our seroprevalences were lower (*T. equi*: 14.3%, *B. caballi*: 2.5%) than those previously reported (*T. equi*: 43.6 and 50.3%, *B. caballi*: 11.4 and 22.2%) [13,14]. Our results were more similar to other recent studies about EP in Spain, one national serosurvey (*T. equi*: 21%, *B. caballi*: 5.6%) [21], and other studies evaluating seroprevalence (*T. equi*: 24.9%, *B. caballi*: 4.6%) in central Spain (Madrid) [22]. In contrast, they were also lower than other old reports in northwestern Spain (Galicia, 2005) [23].

Discrepancies among results could be due to multiple factors. An important difference among studies is the total number of samples evaluated. While approximately 400 horses were included in the previous studies in Andalusia [13,14], 530 in Madrid [22], 580 in the national serosurvey [21], and 60 in Galicia [23], 1002 horses were included in our study. In addition, we collected blood samples during a period of three years, whereas shorter periods (only months) were used in the previous Andalusian studies [13,14]; thus, the effect of different annual climatic conditions on tick distribution and activity cannot be discarded. Another plausible factor influencing the results could be the animal selection criteria used in each study, for example, focusing on horses displaying clinical signs, a specific clinical form, or sampling in farms with previous EP cases. In this sense, a higher seroprevalence was observed in horses displaying EP clinical signs [24] than in those without signs assessed prior to exportation [21]. Moreover, an important bias was observed in the results of the previous Andalusian studies, since donkeys and mules were included in the total seroprevalence calculation [13]. In addition, these donkeys and mules came from only one province (Cadiz) [13], which is one of the provinces with higher EP seroprevalence in Andalusia. Lastly, intrinsic differences inherent to the serologic technique (cELISA versus IFAT versus complement fixation [CF]) used in each study cannot be discarded [13,23]. Noteworthy, the cELISA is considered the most sensitive serological technique and accepted by the WOAH for EP testing [25].

Regarding molecular detection, the true EP prevalence in our study was 10.5%, lower than the results of seroprevalence. However, similarly to serological results, the true prevalence of *T. equi* (27%) was higher than that of *B. caballi* (4.7%) and co-infection (3.3%). Only a previous national survey evaluated *T. equi* and *B. caballi* PCR prevalences in Andalusia [15]. In this study, the results were more comparable with ours (28.7 and 0.8%, respectively), despite only 241 horses being sampled and different PCR techniques being used (multiplex versus singleplex real-time PCR) [15]. Differences between PCR and serologic results could be explained by several factors such as parasitemia levels below the detection limit for the PCR, recent infection leading to low antibody levels undetectable by the cELISA technique, or previous exposure to EP (mainly *T. equi*) causing long-life antibodies in an endemic region [1]. Moreover, the effect of other technical limitations and differences between both techniques cannot be discarded [1].

Geographic localization is an important risk factor influencing EP prevalence [15]. Andalusia is the only Spanish region with both an Atlantic (western) and Mediterranean (eastern) coastline and, although climatically classified as a Mediterranean climate, different weather conditions can be observed, with Atlantic provinces showing higher mean humidity and less extreme mean temperatures than Mediterranean ones [26]. These marked differences within the same region can influence the results and alter the mean prevalence depending on the number of animals included from each province (western versus eastern). Similar patterns have been observed in other regions of Spain, with a higher true prevalence and seroprevalence in western and northern areas of Spain for both parasites [15,21]. The differences between southern and northern areas (mostly *B. caballi*) can also be attributed to rainfall, vegetation abundance, and soil characteristics, which are factors with demonstrated influence on the habitat of the ticks [27,28]. Differences among regions within a country have also been previously reported in other large EP endemic European countries, such as France [29] or Romania [30]. Nonetheless, the effect of the heterogenous number of blood samples submitted from each province cannot be discarded, since a higher number of samples were received from provinces with more registered horses and more high-level sport horses.

EP prevalence in southern European countries (Spain, Portugal, and Italy) [31,32,33] is higher than in northern countries (Ireland, Netherlands, UK, Poland, and Switzerland, among others) [19,34,35,36,37], likely due to climate conditions, vector epidemiology (presence and activity), and husbandry and managements practices [9]. However, although Andalusia is the southernmost EP-endemic region of continental Spain and Europe, the prevalence was lower than those previously reported in other Spanish regions and endemic European countries. This could be related to conditions, such as the low annual mean precipitation and commonly arid environments with low vegetation, not favoring an appropriate ecosystem for vectors despite a Mediterranean climate with a moderate yearly mean temperature. Nonetheless, our findings have some coincidences with other studies performed in southern and endemic European countries, such as southern Romania [38] and Greece [39]. On the other hand, similarly to our study, *T. equi* prevalence was also higher than *B. caballi* in other EP-endemic European countries such as Italy [40], Greece [41], and France [42]. This difference could be explained by the higher capacity of the host immune system to naturally clear *B. caballi* infections, whereas *T. equi* commonly causes a life-long persistent infection [43].

In regard to the prevalence in the provinces within Andalusia, we found higher seroprevalences of *T. equi* in western provinces, although data were lower in our study compared to previous ones [13,14]. Similarly to previous reports, *T. equi* seroprevalence was also higher than that of *B. caballi* in our study, but no differences were observed for *B. caballi* among provinces. In contrast, Garcia-Bocanegra et al. (2013) observed statistical differences between Cadiz (a western province) and Almeria (an eastern one) [13].

This study is the first one evaluating the true prevalence in the provinces within Andalusia; thus, data are not available for comparison. Our results for PCR differ from those for seroprevalence, which was expected due to the lower detection rate of molecular versus serological methods in epidemiological studies [44]. Eastern provinces showed higher prevalences of *T. equi* infection, but this finding was not observed for *B. caballi*. These differences could be attributed to the different number of horses evaluated from each province. Although blood samples submitted from Cordoba, Huelva, and Jaen were lower than those recommended by the power sample calculation, previously available data for Cordoba and Huelva are similar to ours. On the other hand, the high observed prevalence of *T. equi* in Jaen could be misleading, since this province was the one with the lowest number of samples received. Considering that fewer horses (and high-level sport horses) are registered in eastern Andalusia compared to western Andalusia, differences in management, husbandry, and vector control could have influenced these results. Another weakness of our study is the retrospective study design, where missing data regarding background history or the reason to perform molecular or serological testing are unknown. In this sense, whether samples were submitted for pre-exportation or pre-purchase screening or from animals with clinical signs could not be further investigated.

Numerous risk factors have been previously evaluated in EP, such as age, gender, breed, species (mules, donkeys, and horses), regionality, vaccination and deworming status, tick presence, attendance to sport competitions or fairs, etc. Due to the retrospective nature of this study, a multiple logistic regression analysis evaluating these risk factors on EP prevalence in each province could not be performed. A significant positive correlation between age and *T. equi* seropositivity was observed. This finding agrees with previous reports in Andalusia [13], Spain [15], and worldwide [45]. However, this effect was not observed for *B. caballi*, similar to other European studies [9]. This can be due to the higher cumulative risk of pathogen contact and long-lasting seropositivity for *T. equi* in infected horses. Nonetheless, due to missing data, this association between age and *T. equi* seropositivity mut be interpreted with caution.

We did not find a significant effect of sex in our study, similarly to previous ones in Spain [14,22] and Europe [9], although a sex-dependent difference in susceptibility to protozoan infections has been described in horses [46]. Nonetheless, missing values from each province limit the power of this result in our study.

To the authors’ knowledge, this is the first study evaluating the effect of the season and year of testing on EP prevalence in Spain, in particular in Andalusia. Spring and summer presented significantly higher *B. caballi* PCR-positive horses compared to other seasons, but no differences were observed for *T. equi*. In contrast, higher seroprevalences of *T. equi* were observed in spring and autumn. Although anamnesis and clinical data were not collected, these findings could indicate that PCR is the chosen diagnostic method in acute clinical forms, whereas serology is likely preferred for screening in asymptomatic horses (prior to international movement, pre-purchase, or screening within a diagnostic work-up). In this sense, sport and show events are more common in these seasons in Andalusia due to better weather, which also leads to more contact between the owner/rider and horse, facilitating the detection of disturbances such as poor performance. In all these circumstances, EP serology is the diagnostic test choice [1]. A European study did not find differences in seasonality and EP prevalence [9] using both PCR and serology. Since several European countries with different climates were included, the effect of regionality cannot be discarded in this study.

Regarding the year of testing, no differences were observed among years, with *T. equi* prevalence being higher than the prevalence of *B. caballi*, both by PCR and serology, in every year studied. Moreover, more EP-positive horses were detected by serology than by PCR, although size groups were heterogenous. Therefore, from our results, it cannot be concluded that EP prevalence is increasing in Andalusia, at least during this period. Additional studies with a longer period of study could yield more powerful conclusions. Nonetheless, a previous European study evaluating samples from 2008 to 2021 did not observe an effect of year of testing on EP prevalence [9].

The main limitation of this study is the retrospective design, where the history about the purpose of EP testing (exportation, pre-purchase, clinical signs, etc.) or whether clinical signs were observed is unknown. Moreover, some data are missing, for example, regarding sex, breed, or age; thus, a multivariable analysis to control for confounding factors cannot be performed. In addition, because of the retrospective nature, groups size was heterogeneous. Altogether, these shortcomings limit result interpretation.

## 5. Conclusions

EP has widespread prevalence in Andalusia, with western provinces showing different true and seroprevalence than eastern ones (regionality effect). In addition, a seasonality effect was also observed, being different depending on PCR or serology testing. Although Andalusia continues being an EP-endemic region within Spain, lower prevalences (both for PCR and serology) were found compared to previous studies. Moreover, during the studied period, no interannual increase in EP prevalence was observed. In addition, older animals had a higher probability of being *T. equi*-seropositive. Future studies such as a prospective clinical trial or a geographical distribution mapping of infected vectors would provide valuable scientific knowledge.

## Figures and Tables

**Table 1:** Number of horses registered in Andalusia in 2024, grouped by provinces within Andalusia, and number of sampled horses.

	Total Registered Horses	% of Total to Sample	Calculated Number of Horses to Sample	Horses Sampled	% of Total Sampled
**Almeria**	5300	3	30	25	2.5
**Cadiz**	25,399	16	143	187	18.7
**Cordoba**	17,467	11	99	56	5.6
**Granada**	10,413	7	59	85	8.5
**Huelva**	28,651	18	162	102	10.2
**Jaen**	9288	6	52	9	0.9
**Malaga**	19,887	13	112	203	20.3
**Sevilla**	42,633	27	240	335	33.4
**Andalusia**	**159,038**	**100**	**897**	**1002**	**100**

**Table 2:** True prevalence and seroprevalence of *B. caballi* and *T. equi* in Andalusia.

	*B. caballi*	*T. equi*	Co-Infection
**PCR**	4.7% (2.3–7.1%)	27.0% (22.0–32.0%)	3.3% (1.3–5.4%)
**Serology**	2.5% (1.3–3.6%)	14.3% (11.8–16.9%)	1.9% (0.9–2.9%)

Data are expressed as percentage of prevalence and (95% confidence intervals).

## Data Availability

Data are available upon request to the corresponding author.

## Supplementary Materials

The following supporting information can be downloaded at: https://www.mdpi.com/article/10.3390/ani15142047/s1. Table S1: Spearman’s correlation (rho) between contributors and diagnostic methods.
