# Supplementary material for: True Prevalence and Seroprevalence of Piroplasmosis in Horses in Southwestern Europe
Source: Animals (Basel). 2025 Jul 11;15(14):2047. doi: 10.3390/ani15142047 (PMC12291708; doi:10.3390/ani15142047)
Supplement: Supplementary file 1 [file animals-15-02047-s001.zip › animals-3718054-supplementary.pdf]

Table S1. Spearman's rank correlation coefficient (rho) between variables and diagnostic methods.

	**EP PCR positive**		***B. caballi*** **PCR positive**		***T. equi*** **PCR positive**		**EP seropositive**		***B. caballi*** **seropositive**		***T. equi*** **seropositive**	
**Variable**	rho	P value	rho	P value	rho	P value	rho	P value	rho	P value	rho	P value
Season	-0.006	0.92	-0.460	0.43	0.029	0.62	0.051	0.92	-0.022	0.57	0.055	0.15
Year	0.031	0.58	0.037	0.52	0.026	0.65	0.005	0.90	0.031	0.42	-0.016	0.68
Age	-0.145	0.42	-0.126	0.48	-0.145	0.42	0.119	0.03*	0.070	0.22	0.113	0.04*
Gender	0.043	0.81	0.066	0.71	0.043	0.81	0.011	0.85	-0.044	0.44	0.034	0.55

* Statistically significant correlation.
